# Case-Control Study of *Cryptosporidium* Transmission in Bangladeshi Households

**DOI:** 10.1093/cid/ciy593

**Published:** 2018-09-06

**Authors:** Poonum S Korpe, Carol Gilchrist, Cecelia Burkey, Mami Taniuchi, Emtiaz Ahmed, Vikram Madan, Rachel Castillo, Shahnawaz Ahmed, Tuhinur Arju, Masud Alam, Mamun Kabir, Tahmeed Ahmed, William A Petri, Rashidul Haque, A S G Faruque, Priya Duggal

**Affiliations:** 1 Johns Hopkins University Bloomberg School of Public Health, Baltimore, Maryland; 2 University of Virginia School of Medicine, Charlottesville; 3 International Centre for Diarrhoeal Disease Research, Dhaka, Bangladesh

**Keywords:** *Cryptosporidium hominis*, *Cryptosporidium parvum*, *Cryptosporidium meleagridis*, transmission, diarrhea

## Abstract

**Background:**

*Cryptosporidium* is a leading contributor to diarrheal morbidity and mortality in under-5 children worldwide. As there is no vaccine and no effective drug therapy in young children for this infection, preventing infection is critical. We undertook a pilot case-control study to define the extent of person-to-person transmission of cryptosporidiosis within an urban and a rural community in Bangladesh.

**Methods:**

We enrolled 48 case families with a *Cryptosporidium*-infected child aged 6–18 months. Controls were age- and sex-matched *Cryptosporidium*-negative children in 12 households. Children and household members were followed for 8 weeks with weekly illness survey and stool testing with quantitative polymerase chain reaction for *Cryptosporidium*.

**Results:**

In the 24 urban case families, the secondary attack rate was 35.8% (19/53) vs 0% (0/11) in controls (*P* = .018, χ^2^ test). In contrast, in the 24 rural case families, the secondary attack rate was 7.8% (5/64) vs 0% (0/21) in controls (*P* = .19, χ^2^ test). Genotyping by gp60 demonstrated infection with the same subspecies in 5 families, and evidence of transmission in 2. Serologic response to *Cryptosporidium* infection was associated with younger age, longer duration of infection, and *Cryptosporidium hominis* gp60_IbA9G3R2 infection.

**Conclusions:**

In the urban site, the high rate of secondary infection and infection with the same subspecies within families suggests that person-to-person transmission is a major source of *Cryptosporidium* infection for young children living in this region. Molecular genotyping can be applied to determine transmission of *Cryptosporidium* in endemic regions. Further work is needed to understand the differences in parasite transmissibility and immunity to different genotypes.

## Teaser

In this study of *Cryptosporidium* transmission, the secondary attack rate of *Cryptosporidium* was 35.8% in urban households, and family members carried the same *Cryptosporidium* genotype, suggesting that person-to-person infection may be an important source of infection in children.

Globally, cryptosporidiosis is a leading cause of diarrhea in children, and annually there are 2.9–4.7 million *Cryptosporidium*-attributable cases in children <2 years of age in sub-Saharan Africa and South Asia [1, 2]. Both diarrheal and subclinical *Cryptosporidium* infection have been associated with growth faltering and cognitive deficits [3, 4]. Despite the significant public health threat posed by this enteric parasite, there is no effective drug or vaccine for this patient population.

*Cryptosporidium* is transmitted via fecal–oral spread and outbreaks have been associated with contaminated water supplies [5]. In endemic regions, infection is associated with poor sanitation, poverty, animal rearing, and malnutrition [6]. Recent studies aimed at improving household water and sanitation behaviors in Bangladesh showed reduction in overall diarrhea but did not result in decreased *Cryptosporidium* infections or reduction in stunted growth, a known consequence of *Cryptosporidium* [7, 8]. In the absence of effective behavioral and environmental interventions and drug therapies, understanding the factors involved in transmission and acquisition of infection is imperative.

In prior studies, we described *Cryptosporidium* infection rates as high as 80% during the first 2 years of life [9]. However, the route of transmission remains elusive as *Cryptosporidium* species can be transmitted through direct or indirect contact (involving contaminated soil, food, or water). Although water contamination is often the cause of *Cryptosporidium* outbreaks, we hypothesized that in Bangladesh, young children were at greatest risk of infection from household contacts, as >95% of households in this cohort received municipal water, treated by the Dhaka Water and Sewage Authority.

We therefore aimed to understand person-to-person transmission in communities in Bangladesh by tracking the spread of the infection within urban and rural households with young children. The children in these households were of the age range at highest risk for *Cryptosporidium* infection.

## METHODS

### Study Design

We designed a case-control study of families in urban and rural Bangladesh (Bangladesh Household Transmission Study), nested within a larger birth cohort study (ClinicalTrials.gov: NCT02764918). Birth cohorts were established at 2 sites in Bangladesh: a periurban slum within Mirpur, and a rural subdistrict located 60 km northwest of Dhaka, in Mirzapur [10]. In the parent study, children were enrolled at birth, and followed by twice-weekly home visits for diarrheal illness until age 2.

Children were monitored for the first *Cryptosporidium* infection by monthly surveillance stool collection and testing. Children who reached age 6 months without infection were screened on a monthly basis for *Cryptosporidium* infection using a rapid immunodiagnostic test in the field (*Giardia/Cryptosporidium* QUIK CHEK, TechLab), and results were confirmed using a *Cryptosporidium*-specific real-time quantitative polymerase chain reaction (qPCR) assay at the International Centre for Diarrhoeal Disease Research, Bangladesh (icddr,b) Parasitology Laboratory.

Parents of children testing positive for *Cryptosporidium* were consented for enrollment into the Household Transmission Study. All household members, defined as any individual sleeping under the same roof or eating from the same cooking pot, were consented for enrollment. Written consent was obtained from all participants. Index children testing negative were placed into a pool as potential age- and sex-matched community controls. We enrolled 24 case children and 6 age- and sex-matched controls from each site, and their respective household members.

### Surveillance

A baseline demographic survey was collected from each household. Subsequently, all households were visited weekly for 8 weeks, and each subject completed an illness survey querying for diarrhea. A stool specimen was collected from all subjects weekly over 8 weeks. A serum sample was obtained from subjects at weeks 1 and 8.

### Laboratory Testing

Stool specimens were transported to the icddr,b laboratory for *Cryptosporidium* testing using a multiplex qPCR assay [11]. DNA extraction was performed by a modified QiaAmp stool DNA extraction protocol that incorporates a 3-minute bead-beating step to lyse *Cryptosporidium* oocysts (Qiagen, Valencia, California) [11]. All *Cryptosporidium*-positive specimens were speciated using the LIB3 assay to distinguish *Cryptosporidium hominis* from non–*C. hominis* isolates [12]. The polymorphic region within the gp60 gene was used to genotype *Cryptosporidium*-positive samples as previously described [9, 13]. Sera was tested for anti-*Cryptosporidium* immunoglobulin G (IgG), using enzyme-linked immunosorbent assay (ELISA) with whole *Cryptosporidium*-coated plates (2.5 × 10^6^ oocysts/mL) (Waterborne Inc, New Orleans, Louisiana) [14]. Antihuman horseradish peroxidase conjugate was used, and absorbance was measured on the ELISA reader at 450 nm.

### Statistical Analysis

A secondary attack rate was calculated by dividing the number of new infections among household members by the total number of uninfected household members at baseline. A 2-sample *t* test was used to compare differences in means between continuous variables. The χ^2^ test was used to compare categorical variables. Multiple linear regression was used to predict association of IgG responses with independent predictors. Statistical analysis was done using Stata version 13.1 software (StataCorp, College Station, Texas).

### Ethics Approval

The study was approved by the Research and Ethics Review Committees at the icddr,b and by the Institutional Review Board at the Johns Hopkins University Bloomberg School of Public Health. Parents/guardians of all enrolled children provided written consent.

## RESULTS

From August 2015 to February 2017, 60 households were enrolled. This consisted of 24 case families and 6 control families from each site, and included 60 index children and 162 household members. Of the household members, 37% were mothers, 16% were fathers, 28% were siblings, 9% were grandmothers, and the remainder (10%) were grandfathers or other relatives. Of the siblings, 13.8% were aged <5 years.

There were significant differences between the 2 sites (Table 1). The urban site, Mirpur, had a greater percentage of household members testing positive by stool PCR for cryptosporidiosis compared to the rural site, Mirzapur (51% vs 29%; *P* < .01, *t* test). Additionally, there was lower maternal education (mean, 4.5 vs 6.4 years; *P* < .01), more crowding (mean, 4 vs 3 persons/room; *P* < .01), and higher occurrence of toilet sharing in Mirpur compared to Mirzapur (mean, 3.2 vs 1.1; *P* = .03).

**Table 1.** Characteristics of Households From Mirpur and Mirzapur

Characteristic	Urban Mirpur	Rural Mirzapur	*P* Value
No. of case households enrolled	24	24	
No. of household members enrolled	75	87	
No. of children <60 mo of age	1.2	1.2	.5
% of female case children	61	39	.12
% of individuals with *Cryptosporidium*	51	29	<.01
Years of maternal education, mean (SD)	4.5 (2.7)	6.4 (4.0)	<.01
Crowding: No. of persons per room, mean (SD)	4 (0.7)	3 (1.0)	<.01
Monthly income (US dollars), mean (SD)	229 (208)	241(245)	.86
% of homes with any animal	38	100	<.01
Cow	0	54.2	<.01
Goat	16.7	20.8	<.01
Chicken/duck	29.1	79.2	<.01
Water source	100% piped	100% tube well	
% of homes with improved toilet	79	71	.5
No. of households sharing toilet	3.2 (4.5)	1.1 (1.0)	.03

Abbreviations: SD, standard deviation; US, United States.

In Mirpur, 18.9% (n = 154) of weekly surveillance stool samples tested *Cryptosporidium* positive. Of the genotyped samples, 87% were *C. hominis* and 13% were *Cryptosporidium parvum.* In Mirzapur, 10% of weekly surveillance stools tested *Cryptosporidium* positive (n = 94). Of these, 92% were *Cryptosporidium meleagridis*, and 8% *C. hominis.* During the study period, all 24 episodes of diarrhea recorded and stool specimens collected (21 in Mirpur and 3 in Mirzapur) occurred in index children. Twenty-one of the 24 (87.5%) diarrheal episodes were *Cryptosporidium* positive (Mirpur, 18; Mirzapur, 3). In Mirpur, 13 of 18 diarrheal stools were successfully speciated and found to be *C. hominis*. In Mirzapur, 2 of the 3 diarrheal stools were successfully speciated as *C. meleagridis*.

Across both sites there were no infections in the control families (0/32). In the 48 case families, the rate of secondary infection was 20.5% (24/117) overall. In Mirpur, the secondary attack rate was 35.8% (19/53) vs a rate of 7.5% (5/64) in Mirzapur (*P* < .001, χ^2^ test). In 66% (16/24) of case families in Mirpur, multiple family members had concurrent infections with the index child. In 14 of these 24 case families, a second family member who initially tested negative subsequently became positive during the 8-week follow-up period.

Among Mirpur subjects testing positive for *Cryptosporidium*, children aged ≤2 years had a significantly longer period of shedding (mean, 4.1 weeks) compared to individuals aged >2 years (mean, 1.7 weeks) (*P* < .001). Additionally, infants had a higher *Cryptosporidium* parasite burden (lower cycle threshold [Ct]) vs those >2 years of age (mean Ct, 26.3 vs 29.0; *P* = .08).

### GP60 Genotyping Results

**Figure 1.**
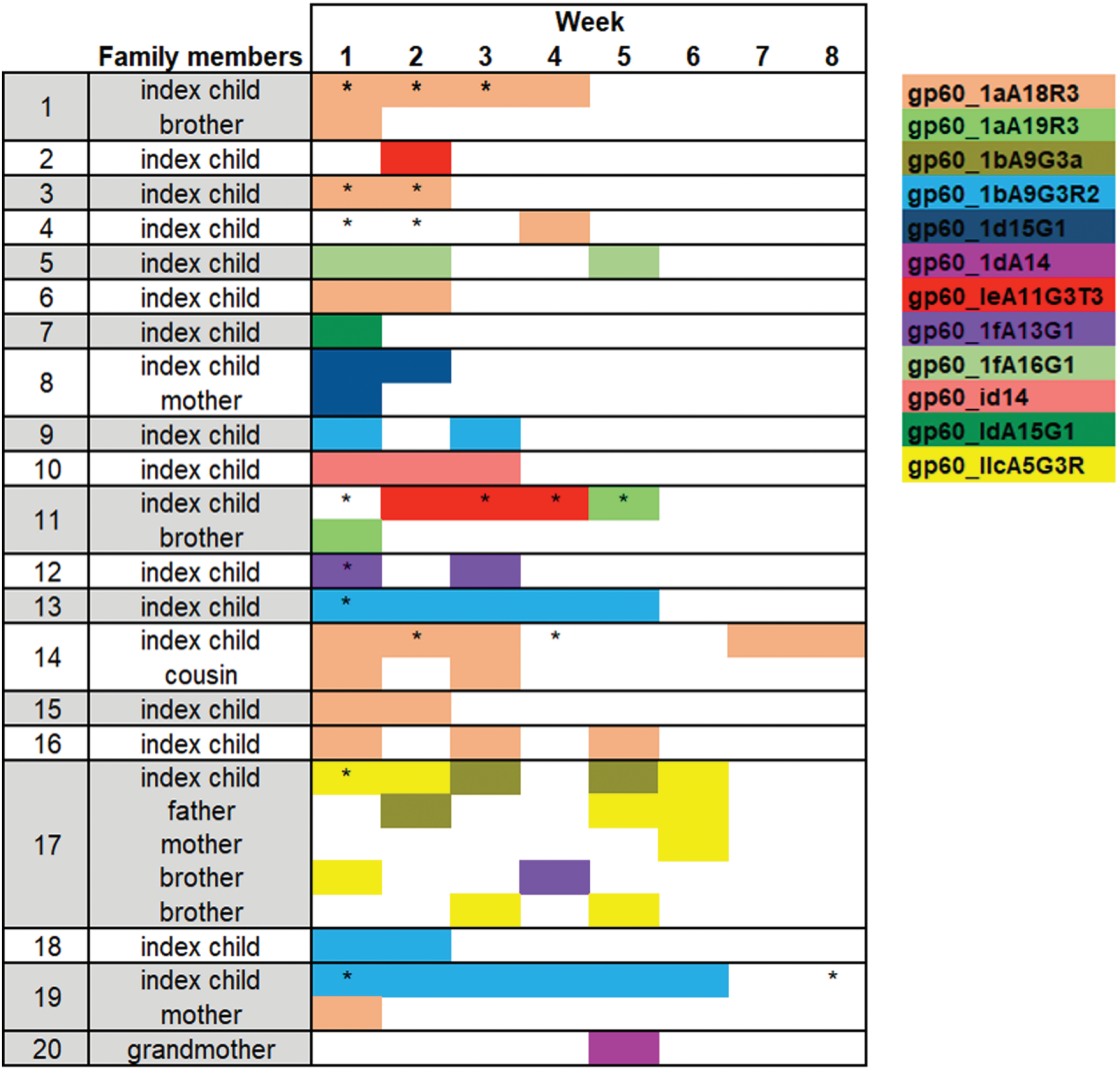
*Cryptosporidium* genotypes detected in 20 Mirpur households over the 8-week follow-up period. A box shaded in color indicates that individual tested positive for *Cryptosporidiu*m in that week and the genotype was identified. An asterisk (*) indicates the subject had *Cryptosporidium*-positive diarrhea in that week, and an asterisk without color indicates positive *Cryptosporidium* detection without identification of genotype. In 6 families, household members were already infected with *Cryptosporidium* at baseline. *Cryptosporidium hominis* gp60_1aA18R3 was the most abundant genotype. Families 11 and 17 demonstrate transmission of a novel genotype from a brother and father to the index child. In individuals with multiple genotypes detected in 1 sample, the most abundant genotype is represented here.

In 8 of 24 Mirpur case families, only the index child tested positive for *Cryptosporidium*, and in 6 of these the *Cryptosporidium* isolate was successfully genotyped; the other 2 had low parasite burden and could not be genotyped. Figure 1 depicts the gp60 genotype of these 6 children.

In 16 of 24 Mirpur families, the index child and at least 1 family member tested positive for *Cryptosporidium*. Four of these could not be genotyped and, in 6 additional families, only the index child was successfully genotyped due to low parasite burden in the other family members. However, in 6 of the 16 families, 2 or more individuals in the household were successfully genotyped (Figure 1). Figure 1 depicts the genotypes and weeks of infection in 20 families.

Overall, we detected 12 distinct *C. hominis* genotypes and 1 *C. parvum* genotype. Genotype *C. hominis* gp60_1aA18R3 was the most abundant genotype in this cohort (n = 9), consistent with the 20% prevalence of this genotype described in the parent study [10, 15]. Additionally, we detected a temporal pattern to infection with certain genotypes. Genotype *C. hominis* gp60_IaA18R3 was present during all 3 years of the study, whereas gp60_IdA15G1 was only detected in 2015, and gp60_IaA19R3 and gp60_IfA13R3 in 2016 (Table 2).

**Table 2.** Subjects With *Cryptosporidium* Genotype and *Cryptosporidium* Immunoglobulin G Antibody Response

Date of Enrollment	Family ID	Subject	Age, y	Genotype	Weeks Genotype Detected	Infection in First 4 Weeks	IgG Weeks 8:1 Ratio
Jan 2016	1	Index	<1	*Cryptosporidium hominis* gp60_IaA18	4	4	1.29
Jan 2016	1	Index	<1	*C. hominis* gp60_IaA17	2	4	1.29
Jan 2016	1	Brother	8	*C. hominis* gp60_IaA18	1	1	0.94
Dec 2015	2	Index	1	*C. hominis* gp60_Ie_A11G3T3_V	1	2	2.24
Sep 2015	3	Index	1	*C. hominis* gp60_IaA18	2	4	3.47
Sep 2015	4	Index	1	*C. hominis* gp60_IaA17	1	…	0.79
Sep 2015	4	Index	1	*C. hominis* gp60_IaA18	1	3	0.79
Jan 2016	5	Index	1	*C. hominis* gp60_If_A16G1	3	4	1.34
Sep 2015	7	Index	<1	*C. hominis* gp60_IdA15G1	1	3	2.17
Sep 2016	8	Index	<1	*C. hominis* gp60_Id15G1	2	2	1.22
Sep 2015	8	Mother	28	*C. hominis* gp60_Id15G1	1	1	1.22
Nov 2015	10	Index	<1	*C. hominis* gp60_IdA14	3	3	1.48
Jun 2016	11	Index	1	*C. hominis* gp60_Ie_A11G3T3	4	4	0.95
Jun 2016	11	Brother	5	*C. hominis* gp60_IaA19	1	1	…
May 2016	12	Index	1	*C. hominis* gp60_IfA13G1	2	4	2.69
Feb 2016	13	Index	1	*C. hominis* gp60_IbA9G3R3_V	5	4	4.03
Nov 2015	14	Index	<1	*C. hominis* gp60_IaA18	5	4	3.32
Nov 2015	14	Cousin	2	*C. hominis* gp60_IaA18	2	2	…
Oct 2015	16	Index	<1	*C. hominis* gp60_IaA18	3	4	4.89
May 2016	17	Index	1	*Cryptosporidium parvum* gp60_IIcA5G3R2	4	3	1.98
May 2016	17	Father	32	*C. parvum* gp60_IIcA5G3R2	3	2	1.41
May 2016	17	Brother	5	*C. parvum* gp60_IIcA5G3R2	2	1	…
May 2016	17	Brother	6	*C. parvum* gp60_IIcA5G3R2	1	3	…
May 2016	17	Mother	24	*C. parvum* gp60_IIcA5G3R2	1	3	1.84
Feb 2016	18	Mother	30	*Cryptosporidium hominis* gp60_IaA18	1	2	…
Feb 2016	19	Index	<1	*C. hominis* gp60_IbA9G3R2	6	4	7.85

Included in this table are subjects who had stool samples successfully genotyped and had serum IgG results. Children aged ≤2 years had the most robust increase in IgG from week 1 to week 8. In general, subjects with greater weeks of stool positivity had the most robust response (index children 13, 14, and 19). *Cryptosporidium hominis* gp60_IaA18 was the most commonly detected in this cohort and was associated with a positive IgG response. Only 1 family had infection with *C. parvum* gp60_IIcA5G3R2; all individuals tested in this family had a positive serologic response. Index child 11 tested positive for *C. hominis* gp60_Ie_A11G3T3 for 4 weeks but failed to show a serologic response.

Abbreviations: ID, identifier; IgG, immunoglobulin G.

In families with multiple members genotyped, we observed concurrent infection with the same genotype in 4 of 6 families (families 1, 8, 14, 17) and thus we could not determine directionality of the transmission at entry into the study. In the sixth family (family 19), the index child was persistently positive for 6 weeks with genotype gp60_IbA9G3R2, whereas the mother was positive with gp60_IAa18R3 in week 1, and never transmitted this to the child and did not pick up the child’s genotype despite the persistent shedding. In family 11, the index child was persistently positive with genotype gp60_IeA11G3T3_V in weeks 2–4 of the study, then in week 5 became positive with gp60_1aA19, the same genotype that her 5-year-old brother was shedding in week 1.

Family 17 was the only family in which *C. parvum* was isolated. In this family, in week 1, both the index child and 6-year-old brother were shedding *C. parvum* IIcA5G3R2. The index child had a concurrent infection with *C. hominis* gp60_IbA5G3R3. In week 2, the father became infected with *C. parvum* IIcA5G3R2, and in week 3, a 5-year-old brother also became infected with the same genotype. The younger brother and father remained infected in week 5, and then in week 6, the mother also became infected with the *C. parvum* genotype.

### Serologic Response

Anti-*Cryptosporidium* IgG was measured at weeks 1 and 8 in 34 individuals in Mirpur (17 children aged ≤2 years, and 17 subjects aged >2 years). There was a greater mean increase in IgG level from week 1 to week 8 in children ≤2 years vs older subjects (mean difference, 0.82 vs 0.32; *P* = .016). A higher week 1 IgG level was associated with fewer weeks of *Cryptosporidium* positivity during the follow-up period when adjusting for age (linear regression; β = –0.85 [95% confidence interval {CI}, –1.5 to –.18]).

The serologic response in week 8 was associated with the number of weeks *Cryptosporidium* was detected in stool during the 8-week period when adjusting for age (β = 0.11 [95% CI, .0–.23]). Genotype *C. hominis* gp60_IbA9G3R2 was significantly associated with a high IgG response when adjusting for weeks of positivity and sex (linear regression; β = 5.37 [95% CI, 1.1–9.6]). Genotype *C. hominis* gp60_IaA18 was the most common genotype isolated and appeared to be consistently associated with a positive serologic response (Table 2). Genotype *C. hominis* gp60_IeA11G3T3 was seen in 1 index child, and despite the child shedding for 4 weeks, there was no serologic response to this genotype.

## DISCUSSION

This is the first study to use molecular diagnostics to evaluate and describe person-to-person transmission of cryptosporidiosis to the subspecies level in an endemic region. Our study describes circulating *Cryptosporidium* genotypes, duration of infection, and development of a serologic response in the setting of subclinical infection. We found significant differences in the rate of household infection and circulating *Cryptosporidium* species between the urban and rural site.

We describe a high rate of *Cryptosporidium* infection among persons of all ages in Mirpur households, similar to that reported in a Brazilian study, where 39% of households had a secondary infection in a household contact [16]. However, after applying gp60 genotyping, we were unable to establish directionality of transmission in a majority of cases, due to our study design. We recruited families based on a child already testing positive for *Cryptosporidium* at the start of the study, so new infections were only studied in relation to the index child. Genotyping results demonstrated that the index child was not transmitting infection to other family members. In this cohort, genotyping revealed 13 distinct subspecies, and we noted multiple species and subspecies of *Cryptosporidium* circulating within a household; in 6 households, multiple individuals were infected with the same *Cryptosporidium* subspecies at entry into the study, which could reflect person-to-person transmission. We noted 2 examples of an older household member, a brother and father, transmitting a novel *Cryptosporidium* infection to the index child.

Though we were unable to establish directionality of transmission in most households, we noted a high rate of new infections among family members in Mirpur, but not in rural Mirzapur. The low rate of secondary infections in Mirzapur could be explained by differences in predominant species at the different sites. We previously reported *C. meleagridis* in 90% of samples collected from Mirzapur, whereas *C. hominis* was the most common species in Mirpur. In the present study, 92% of speciated samples from Mirzapur were *C. meleagridis* [9, 10]. Avian species have been identified as the natural reservoir for *C. meleagridis*, and zoonotic transmission has been described from domesticated chickens to humans [17]. In our study, 6 of the 8 families with *C. meleagridis* infection kept chickens at home, suggesting zoonotic transmission. In contrast, most infections in the urban site were attributable to *C. hominis*, for which humans are the only natural host. Thus, the differences in rates of infection may be directly due to different transmission modes of predominant *Cryptosporidium* species in these 2 different regions. Additionally, it is known that the infectious dose of *Cryptosporidium* can vary from 10 to 1000 oocysts, depending on the species and strain [18, 19]. Thus, it is possible that in Mirpur, the higher secondary attack rate is a direct result of a lower infectious dose of *C. hominis* vs that of *C. meleagridis*, which was predominant in the rural site.

*Cryptosporidium hominis* gp60_1aA18R3 was the most frequently isolated genotype, and mostly noted in concurrently infected individuals in the same household. It is difficult to say whether this genotype is more prevalent because it is environmentally ubiquitous, or because it is more easily transmitted, either due to low infectious dose or other pathogenic characteristics of the parasite.

In 2 households that were completely genotyped, we observed that infection started in 1 individual and then spread to other individuals, which we propose is evidence for person-to-person transmission. In 1 family, we documented transmission from an older sibling to the index child. And in the second family, the only family with *C. parvum* infection, we documented transmission from children to parents and then to other children. Notably, we did not document any other cases of child-to-adult transmission, presumably because adults had acquired immunity. We have previously documented that *C. hominis* is the dominant circulating species in Mirpur, and likely most older subjects have been exposed to *C. hominis* previously in life; in contrast, *C. parvum* has previously been documented in only 2% of infections in this area [9, 10]. One possibility is that the *C. parvum* genotype currently isolated was newer to this area and, potentially, even adults had not previously developed immunity. This would explain why the *C. parvum* IIcA5G3R2 genotype was so easily transmitted between individuals of all ages in this household.

We found that the first *Cryptosporidium* infection may be the most important in stimulating a serologic response, as children with their first infection had the most robust increase in IgG over the 8-week follow-up period vs older subjects. This is consistent with a study from India, which showed that IgG response peaked 9 weeks after a *Cryptosporidium* diarrheal episode in children [20]. However, our findings are novel as we detected a serologic response even in subclinical, nondiarrheal infections [21, 22]. We also noted that higher IgG level at baseline was associated with fewer weeks of *Cryptosporidium* positivity, implying that elevated IgG may be correlated with an effective immune response. In subjects older than 2 years, the average duration of shedding was significantly lower compared with younger children. This likely reflects greater immunity; even if older individuals become infected, they may be better able to control infection and have less shedding of the parasite, as reflected by the lower parasite burden we found in older individuals.

Prior studies have noted immunologic cross-reactivity with infection from different *Cryptosporidium* species. One study in Bangladesh demonstrated serologic response to a *C. parvum* gp15 antigen in children with symptomatic *C. hominis* disease [23]. In our study, we used whole *C. parvum* oocyst as the antigen in the ELISA, and even though most infections were due to *C. hominis*, we found a robust IgG response in children infected with *C. hominis* subtypes. *Cryptosporidium hominis* gp60_IbA9G3R2 was associated with a particularly strong response. Conversely, in a case with *C. hominis* gp60_IeA11G3T3, despite persistent infection for 4 weeks, the child failed to demonstrate a serologic response. Our results suggest that there may be species- and subspecies-level cross-reactivity in immunogenic responses in children, which warrants further study.

During this period of intense surveillance, we detected 24 diarrheal episodes, 21 of which were *Cryptosporidium* associated, and all occurred in index children. This is consistent with our prior study, which found high rates of nondiarrheal *Cryptosporidium* infection in Bangladesh [9]. There was no association between diarrheal infection and transmission. This highlights the fact that in households with small children, multiple individuals have subclinical *Cryptosporidium* infection, and even though they lack symptoms, they may be silently shedding the parasite and spreading infection.

Limitations of this study include the sample size, as this was a pilot study. We successfully demonstrated that adults and children within this community could be intensively and longitudinally studied, and our findings can now be followed up in a larger study. Second, we were unable to genotype all *Cryptosporidium*-positive stool samples, likely due to the low parasite burden in some stool specimens, primarily in adults. Last, we lacked sampling and testing of environmental samples, such as drinking water and soil, which could have helped to establish environmental burden.

Our study offers unique insight into the endemic transmission of cryptosporidiosis, a disease without vaccine or effective therapy in children. Cryptosporidiosis is known to be spread by the fecal–oral route, and our findings of higher prevalence in areas with more overcrowding and toilet sharing are consistent with this paradigm. The recent WASH Benefits Bangladesh Trial, a cluster randomized study of clean water, handwashing, and sanitation intervention, did not find any decrease in cryptosporidiosis in children receiving improved water and sanitation [8]. Another Indian study also failed to show reduction in cryptosporidiosis with clean drinking water [24]. Coupled with these results, our findings suggest that even if water and sanitation interventions achieve high adherence, children still acquire cryptosporidiosis, likely from other infected individuals. Humans are the only natural host for anthroponotic species of *Cryptosporidium*, so further work is needed to understand determinants of transmissibility of the parasite, including whether age and acquired immunity impact oocyst shedding and infectivity. Ultimately, tackling cryptosporidiosis in endemic regions will require an improved understanding of routes of transmission to young children, as well as concerted efforts at identifying environmental reservoirs of the parasites, and understanding differences in pathogenicity of different species and subspecies.

## Notes

***Acknowledgments.*** We thank the children and families of Mirpur and Mirzapur for their participation in this study.

***Financial support.*** This work was supported by the Bill & Melinda Gates Foundation (grant number OPP1127192 to P. K.); the National Institutes of Health (grant numbers K23 AI108790 to P. K. and R01 AI043596 to W. A. P.); and the Sherrilyn and Ken Fisher Center for Environmental Infectious Diseases Discovery Program (to P. D.).

***Potential conflicts of interest.*** W. A. P. has received personal fees from TechLab, Inc. All other authors report no potential conflicts of interest. All authors have submitted the ICMJE Form for Disclosure of Potential Conflicts of Interest. Conflicts that the editors consider relevant to the content of the manuscript have been disclosed.
